# Obeticholic Acid Inhibits Anxiety via Alleviating Gut Microbiota-Mediated Microglia Accumulation in the Brain of High-Fat High-Sugar Diet Mice

**DOI:** 10.3390/nu13030940

**Published:** 2021-03-15

**Authors:** Li Wu, Yuqiu Han, Zhipeng Zheng, Shuai Zhu, Jun Chen, Yuanyuan Yao, Siqing Yue, Andreas Teufel, Honglei Weng, Lanjuan Li, Baohong Wang

**Affiliations:** 1 State Key Laboratory for Diagnosis and Treatment of Infectious Diseases, National Clinical Research Center for Infectious Diseases, Collaborative Innovation Center for Diagnosis and Treatment of Infectious Diseases, The First Affiliated Hospital, Zhejiang University School of Medicine, Hangzhou 310003, China; cfightive@zju.edu.cn (L.W.); 11918221@zju.edu.cn (Y.H.); zpzheng@zju.edu.cn (Z.Z.); 11818164@zju.edu.cn (S.Z.); 21918242@zju.edu.cn (J.C.); yaoyuanyuan0212@zju.edu.cn (Y.Y.); ljli@zju.edu.cn (L.L.); 2 Research Units of Infectious Disease and Microecology, Chinese Academy of Medical Sciences, Hangzhou 310003, China; 3 Key Laboratory of Microbial Technology for Industrial Pollution Control of Zhejiang Province, College of Environment, Research Center of Environmental Science, Zhejiang University of Technology, Hangzhou 310032, China; yuesiqing@126.com; 4 Department of Medicine II, Division of Hepatology, University Medical Center Mannheim, Medical Faculty Mannheim, Heidelberg University, 68167 Mannheim, Germany; andreas.teufel@medma.uni-heidelberg.de (A.T.); Honglei.Weng@medma.uni-heidelberg.de (H.W.)

**Keywords:** obeticholic acid, anxiety, gut microbiota, neuroinflammation, bile acids, metabolic disorders

## Abstract

Anxiety is one of the complications of metabolic disorders (MDs). Obeticholic acid (OCA), the bile acids (BAs) derivative, is a promising agent for improving MDs in association with gut dysbiosis. Yet, its protective effect on MDs-driven anxiety remains unknown. Here, we assessed the serum biochemical parameters and behavioral performance by open field and Morris water maze tests in HFHS diet-induced MDs mice after OCA intervention for nine and 18 weeks. Moreover, antibiotics intervention for microbial depletion was conducted simultaneously. We found that OCA treatment inhibited the initiation and progression of anxiety in HFHS diet-MDs mice via a microbiota–BAs–brain axis: OCA decreased the neuroinflammatory microglia and IL-1β expression in the hippocampus, reversed intestinal barrier dysfunction and serum proinflammatory LPS to a normal level, modified the microbial community, including the known anxiety-related Rikenellaceae and *Alistipes*, and improved the microbial metabolites especially the increased BAs in feces and circulation. Moreover, the OCA-reversed bile acid taurocholate linked disordered serum lipid metabolites and indole derivatives to anxiety as assessed by network analysis. Additionally, microbial depletion with antibiotics also improved the anxiety, microgliosis and BAs enrichment in the experimental MDs mice. Together, these findings provide microbiota–BAs–brain axis as a novel therapeutic target for MDs-associated neuropsychiatric disorders.

## 1. Introduction

Anxiety is the most prevalent mental health condition affecting 10.4% of the Western world population [1,2] and predicts later neuropsychiatric disorders, including cognitive impairment [1,3]. Currently, the available therapeutic drugs for anxiety targeting the central nervous system (CNS) showed many side effects, such as sexual dysfunction, persistent hypertension and metabolic disorders (MDs) [4]. However, the underlying pathogenesis of anxiety is poorly understood. Metabolic disorders (MDs) are recognized as a risk factor for neuropsychiatric diseases, including anxiety recently [5,6]. Preliminary investigations evidenced that intestinal microbiota (IM) and its metabolites were involved in both MDs [7,8] and neuropsychiatric diseases [9,10], indicating a potential role of IM in the development of MDs-associated anxiety. Until now, studies on the correlation among IM, MDs, and anxiety are still lacking.

IM has been implicated as a key player in the gut–brain axis and regulates host brain function and behavior [11]. More recently, the association between IM and MDs-related neuropsychiatric dysfunction has been reported. For example, mice receiving fecal bacteria from cognitive dysfunction diabetic mice showed cognitive impairment [12]. Additionally, regulation of commensal bacteria could restore cognitive function in high-fat diet (HFD)-induced obese mice [13]. These studies highly demonstrated the link between IM and MDs-related cognitive impairment. As a predictor of cognitive dysfunction, however, few studies reported the role of IM in anxiety, which was associated with MDs.

It is well-established that bile acids (BAs) act as critical microbial metabolites to interact with the host physiology and trigger responses in both the local organs and even the distant brain [14,15,16]. BAs were recognized as important signaling molecules in regulating lipid and glucose metabolism [17]. Recently, the epidemiological study supported that the BAs were involved in the pathogenesis of neuropsychiatric diseases, such as Alzheimer’s disease [9]. In addition, studies have indicated the correlation between BAs and anxiety. For example, the cholestasis mice with elevated serum BAs displayed anxiety-like behavior [18]. Interestingly, the inhibition of BAs reabsorption decreased anxiety-like behavior in nonalcoholic steatohepatitis (NASH)-induced hepatocellular carcinoma mice [19]. Thus, we hypothesized that IM-mediated-BAs might play roles in MDs-related anxiety.

Obeticholic acid (OCA), a synthetic bile acid derivative, was used in clinical trials to treat MDs, including type 2 diabetes mellitus and nonalcoholic fatty liver disease and could improve glucose and lipid metabolism [20]. However, the neuroprotective effects of OCA on MDs-related anxiety remain unknown. Here, we examined the impact of OCA on anxiety in MDs mice induced by a high-fat, high-sugar (HFHS) diet and explored the underlying mechanisms. The gut microbiota, metabolomics, serum biochemical parameters, neuroinflammation, intestinal permeability, and endotoxemia combined with conventional behavioral tests were conducted. The commensal eradicating-antibiotics (ABX) was also used to determine the role of gut microbiota in MDs-related anxiety. Our study suggested the neuroprotective effect of OCA/microbiota on MDs-related anxiety, highlighting the IM and BAs as novel promising targets for metabolism-implicated neuropsychiatric diseases.

## 2. Materials and Methods

### 2.1. Animal Experiment

The experiment procedures were approved by the Animal Ethics Committee of the First Affiliated Hospital, College of Medicine, Zhejiang University. Male C57BL/6 mice (six-week-old, 16–18 g) were purchased from SLAC Laboratory Animal Ltd., Co. (Shanghai, China). All mice were maintained in a specific pathogen-free facility with free access to diet and water and acclimated in one cage for one week. The body weight and serum biochemistry parameters, including fasting glucose, triglyceride (TG), total cholesterol (TC), alanine aminotransferase (ALT) and aspartate transaminase (AST), were examined using Hitachi 7600 automatic biochemical analyzer (Hitachi, Tokyo, Japan)before the experiment and used for grouping to ensure uniformity among groups.

As shown in Figure S1, first, 32 mice were fed with HFHS diet (fat 60% kcal, carbohydrate 20% kcal, protein 20% kcal (Research Diets, New Brunswick, NJ, USA) and carbohydrates (18.9 g/L sucrose and 23.1 g/L fructose) in drinking water) [21] for nine weeks to induce MDs and 12 mice were fed with normal chow. Then HFHS diet mice were divided into three groups (*n* = 10–12 per group): (1) MO; mice were daily oral gavage with OCA (Selleck, USA) (2 mg/mL in carboxymethyl cellulose (CMC-Na), 5 mL/kg) [22]; (2) MA, mice were daily oral gavage with antibiotics cocktail containing1.86 mg ampicillin, 1.86 mg neomycin sulfate, 1.2 mg metronidazole and 0.96 mg vancomycin (Sigma-Aldrich, St. Louis, MO, USA) in 300 μL double distilled water [23]; (3) M, mice were oral gavage with an equal volume of CMC-Na. Meanwhile, normal chow mice were oral gavage with an equal volume of CMC-Na as the control group (C). All the mice were anesthetized and sacrificed at the end of the 27th week.

### 2.2. Measurement of Serum Biochemical Parameters

Fasting blood samples were collected at the sixth, 16th and 24th week and centrifugated at 3500 rpm for 15 min (Eppendorf, Hamburg, Germany) to obtain the serum. The serum levels of fasting glucose, TG, TC, ALT and AST were measured using Hitachi 7600 automatic biochemical analyzer (Hitachi, Tokyo, Japan) [24].

### 2.3. Glucose Tolerance Test

The glucose tolerance tests (GTT) were performed at the eighth, 17th and 25th weeks of the experiment. Briefly, the mice following six hours of fasting were injected with glucose at time zero, and blood glucose levels were examined at five time points, including zero, 15, 30, 60, and 120 min after injection using an Accu-Chek glucometer (Roche, Basel, Switzerland). The area under the curve (AUC) of GTT was calculated to reflect the difference of glucose excursion curves among groups [24].

### 2.4. Histological Examination of the Liver

Fresh liver samples were fixed immediately in 10% neutral buffered formalin and embedded with paraffin. The paraffin-embedded liver sections were stained with hematoxylin and eosin (H&E) and assessed by an experienced pathologist using the NAFLD activity score system in a blinded fashion [25].

### 2.5. Morris Water Maze Test

To assess the cognitive function of mice, the Morris water maze (MWM) experiments were performed at the 18th and 27th weeks. As previously described [26,27], the MWM task was performed in a circular tank (90 cm diameter) with a movable platform (7 cm diameter). Before the experiment, the water was warmed to about 22 °C, and the nontoxic titanium white-colored dye was added to make the water opaque. During the training experiments in five consecutive days, mice learned to find the hidden platform in the center of the target quadrant, which was submerged 1 cm under the water and stayed on it for 15 s before removed. The mice that could not locate the platform within 60 s were put on it and allowed to stay for 15 s. The training experiment was performed four trails per day to assess the spatial learning, and the time to find the platform (escape latency) was measured and averaged for statistical analysis. A probe test was conducted immediately on the sixth day to assess the spatial memory, and the platform was removed to record the time spent in the targeted quadrant.

### 2.6. Open Field Test

Open field (OF) test was used to assess the locomotor activity and anxiety-like behavior of mice. Briefly, mice were gently placed in the center of a plastic open field arena (50 cm × 50 cm × 50 cm) in a dark room with sound-insulated and allowed to explore freely for 10 min. A digital camera connected to a computer positioned above the arena and was used for tracking the total distance and the amount of time spent in the center. The total distance and mean speed were calculated to reflect the locomotor activity, and more time spent in the edges of the box and less in the center were interpreted as anxiety-like behavior [4,26].

### 2.7. Intestinal Permeability In Vivo

The intestinal permeability was assessed using an in vivo fluorescein isothiocyanate (FITC)-dextran (Sigma-Aldrich, St. Louis, MO, USA) as previously described [28]. Briefly, the mice were deprived of food and water for four hours and administrated with FITC-dextran (600 mg/kg body weight, 120 mg/mL). The plasma samples were collected after four hours, and fluorescence intensity was measured using a fluorescence spectrophotometer (Thermo Fisher Scientific, Waltham, MA, USA) at an excitation/emission wavelength of 485 nm/535 nm. The FITC-dextran concentrations were determined using a standard curve generated with a serial dilution of FITC-dextran nontreated plasma with PBS.

### 2.8. Measurement of Serum Lipopolysaccharide Level

The serum lipopolysaccharide (LPS) levels were measured using limulus amoebocyte lysate (LAL) chromogenic endpoint assay (Hycult Biotech, Uden, The Netherlands). Briefly, the serum samples were diluted 1:3 with endotoxin-free water and heated at 75 °C for five minutes in a water bath to neutralize the endotoxin inhibiting compounds. Then, the LAL reagent was added and incubated with the samples for 20 min at 25 °C. Finally, the reactions were terminated by adding the stop solution, and the samples were measured by spectrophotometer (Biotek, Winooski, VT, USA) with the absorbance at 405 nm [29].

### 2.9. Immunofluorescence Staining of Microglial Cells in the Hippocampus

The hippocampus was carefully removed and fixed immediately in 10% neutral buffered formalin, then embedded with paraffin. The paraffin-embedded hippocampus was cut into 4 µm sections for immunofluorescence staining as previously described [30]. The Iba-1 antibody (Servicebio, Wuhan, China) was stained to detect microglial cells, followed by secondary antibodies of CY-3-conjugated goat anti-rabbit IgG (Servicebio, Wuhan, China). Moreover, the DAPI (Servicebio, Wuhan, China) was used for nuclear staining. The Image-Pro Plus 3.0 software (Media Cybernetics, Silver Spring, MD, USA) was used to mark cells in 6 randomly selected 200× fields [31,32] and the positive cells were quantified manually [33].

### 2.10. qPCR Analysis of Proinflammatory Cytokines in the Hippocampus

Total RNA was isolated from the hippocampus using RNeasy Plus mini kit (Qiagen, Hilden, Germany) according to the manufacturer’s protocol. Then the reverse transcription was accomplished by QuantiTect reverse transcription kit (Qiagen, Hilden, Germany). The relative mRNA expression was measured using QuantiFast SYBP green PCR kit (Qiagen, Hilden, Germany) in ViiA7 real-time PCR system (Applied Biosystems, Foster City, CA, USA) [34], and the primer sequences used for amplification are shown in Table S1. The expression of each gene was normalized to β-actin expression, and results were calculated using the comparative cycle threshold (CT) method [30].

### 2.11. The Bacterial 16S rRNA Gene Sequencing

The genomic DNA was extracted from colon contents using a DNA extraction kit (QIAGEN, Hilden, Germany) according to the manufacturer’s instructions, and the concentration and quality of the DNA were determined by Nanodrop 2000 spectrophotometer (Thermo Fisher Scientific, USA) and 1.0% agarose gel electrophoresis, respectively [24]. Then, the genomic DNA was used as templates for PCR amplification of 16S rRNA V3-V4 region with a forward primer (5′-CAAGCAGAAGACGGCATACGAGATGTGACTGGAGTTCAGACGTGTGCTCTTCCGATCT-3′) and reverse primer (5′-AATGATACGGCGACCACCGAGATCTACACTCTTTCCCTACACGACGCTCTTCCGATCT-3′). Next, the equimolar PCR products were pooled, and sequencing was performed in the Illumina NovaSeq platform. The sequences with 97% similarity were clustered into operational taxonomic units (OTU), and OTU tables with Greengenes identifiers were generated in QIIME analysis systems [35]. The statistical difference of α-diversity reflecting community richness (Chao1 and ACE) and community diversity (Shannon and Simpson index) [36] were determined by one-way ANOVA or Kruskal–Wallis test. The PERMANOVA (Adonis) was used to evaluate β-diversity (principal coordinates analysis (PCoA)) based on Bray–Curtis distance matrices [10]. The respective PERMANOVA R values showed community variation between groups, and *p* < 0.05 indicated significance [10].

### 2.12. High-Throughput Untargeted Metabolomics Profiling

#### 2.12.1. Chemical Reagents

HPLC grade methanol, acetonitrile, formic acid and ultrahigh quality water were purchased from Thermo Fisher Scientific (Waltham, MA, USA).

#### 2.12.2. Sample Preparation

The fresh fecal samples were collected at the 27th week and stored at −80 °C until analysis. The fecal samples were prepared by mixed with methanol at a ratio of 3 mL/g [37] and homogenized (8 m/s, 15 s) by adding ceramic beads (1 mm, Omni International, Bedford, NH, USA). The mixtures were centrifuged (12,000 rpm, 10 min, 4 °C) (Eppendorf, Hamburg, Germany), and the supernatants were filtered using 0.22 μm syringe filters (Millipore Corp, Billerica, MA, USA). The serum samples were extracted by mixing with acetonitrile at a ratio of 1:3 and vortexed immediately to remove protein [38]. The mixtures were then centrifuged (12,000 rpm, 10 min, 4 °C) to obtain the supernatants for processing. The procedural blank sample of water was used to monitor the contamination during sample preparation. The quality control (QC) samples of all mixing extraction were processed before and during sample processing to ensure the quality of metabolic profiling [38].

#### 2.12.3. Untargeted Metabolomics Profiling

The metabolomics profiling was analyzed in the Dionex UltiMate 3000 RS ultraperformance liquid chromatography (UPLC) system coupled to Orbitrap Q exactive mass spectrometry (MS) (Thermo Fisher Scientific, Waltham, MA, USA). A Hypersil Gold C-18 column (2.1 × 100 mm, 1.9 µm, Thermo Fisher Scientific, Waltham, MA, USA) was used for compound separation at 35 °C. The eluents employed in both electrospray ionization-positive (ESI +) and negative model (ESI -) were 2–98% gradient of H_2_O-methanol, and 0.1% formic acid was added as an additive. The mass spectral data are acquired by a full MS (range 70–1050 *m*/*z*) followed by data-dependent MS/MS scans. The resolution of Orbitrap was set at 60,000 in full MS scan, and the MS-MS data are collected at the collision energy of 20, 40 and 60 eV.

#### 2.12.4. Data Analysis

First, the raw data are acquired by Xcalibur 4.1 software (Thermo Fisher Scientific, Waltham, MA, USA), and initial data processing, including peak detection and peak alignment, was performed in Compound Discoverer 3.1 software (Thermo Fisher Scientific, Waltham, MA, USA) as described previously [39]. Then, the partial least-squares-latent structure discriminates analysis (PLS-DA) models were built for multivariate statistical analysis using SIMCA-P 13.0 (Umetrics AB, Umea, Sweden) and metabolites with variable importance in the project (VIP) values of more than 1 were selected as differential metabolites [40]. Next, univariate analysis was performed, and the biologically significant metabolites were identified according to the pathway mapping results in Compound Discoverer 3.1 by referring to local libraries and online databases, among which the online data sources of ChemSpider were composed of the Human Metabolome Database, Kyoto Encyclopedia of Genes and Genomes, and Biocyc. Moreover, the mzCloud library was also used for spectral searching. Finally, the hierarchical cluster analysis of biological differential metabolites was conducted in Cluster 3.0 software [41].

### 2.13. Targeted Profiling of Bile Acids

The mass list, including 30 BAs (Table S2), was compiled by the “search mass lists” node of the Compound Discoverer 3.1, using the full-MS scan to pick out all detected BAs metabolites from feces and serum samples of mice.

### 2.14. Statistical Analysis

The statistical analysis was performed using one-way ANOVA or Kruskal–Wallis test by SPSS software (SPSS Inc., Chicago, IL, USA) and GraphPad Prism (GraphPad Software, Inc., La Jolla, CA, USA). Moreover, *p* < 0.05 was considered significant. The correlation analysis was conducted by the Pearson’s or Spearman rank correlation analysis, and the correlation coefficients with *p* < 0.05 were used to build a correlation network in the Cytoscape software package [38].

## 3. Results

### 3.1. OCA Supplementation Inhibited Gut Microbiota-Mediated Anxiety in HFHS Diet-MDs Mice

Mice with HFHS diet showed significantly increased body weight compared with normal chow mice since the first week (Figure S2A). The biochemical parameters that reflect lipid disorders, including TC and TG, were increased in HFHS diet mice compared with normal chow mice (Figure S2B). The GTT results showed that the fasting serum glucose and glucose levels at 30, 60 and 120 min after glucose injection were significantly higher in HFHS diet mice than those with normal chow (Figure S2C). Moreover, the AUC of GTT was also significantly increased in mice with an HFHS diet (Figure S2D). These findings evidenced that mice with a nine-week HFHS diet developed MDs.

**Figure 1:**
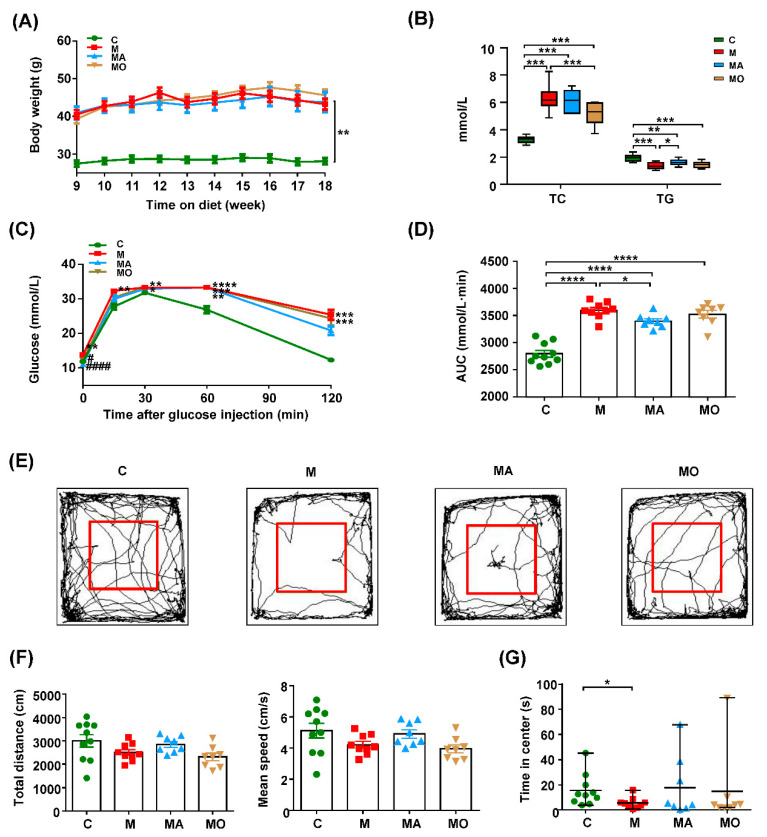
Obeticholic acid (OCA) supplementation inhibited the anxiety of high-fat, high-sugar (HFHS) diet-induced metabolic disorders (MDs) mice in association with gut microbiota. HFHS diet-induced MDs mice showed anxiety-like behavior, which was inhibited by OCA or eradicating-antibiotics (ABX) supplementation. (**A**–**D**) The body weight, serum total cholesterol (TC) and triglyceride (TG), glucose tolerance tests (GTT) and the corresponding area under the curve (AUC) were evaluated at the 18th week. (**E**) The representative trace graphs of the OF test at the 18th week. The parameters of the OF test reflecting (**F**) locomotor activity and (**G**) anxiety-like behavior are depicted. Note: data are given as mean± SEM or medians with range. *n*: (**A**–**G**) 8–12 per group. (**A**,**C**) * *p* < 0.05; ** *p* < 0.01; *** *p* < 0.001 and **** *p* < 0.0001 compared with normal chow mice, # *p* < 0.05; #### *p* < 0.0001 compared with HFHS diet mice with vehicle. (**B**,**D**,**G**) * *p* < 0.05; ** *p* < 0.01; *** *p* < 0.001; **** *p* < 0.0001. Groups: C, mice with normal chow; M, mice with HFHS diet; MA, ABX-treated HFHS diet mice; MO, OCA-treated HFHS diet mice. Abbreviations: HFHS, high-fat high-sugar; MDs, metabolic disorders; ABX, antibiotics; OCA, obeticholic acid; TC, total cholesterol; TG, triglyceride; GTT, glucose tolerance test; AUC, area under the curve; SEM, standard error of mean; OF, open field.

To explore the role of the microbiota–BAs axis in the development of MDs and the related anxiety, the synthetic bile acid derivative OCA and commensal eradicating ABX were administrated to HFHS diet mice since the ninth week. As shown in Figure 1A, the bodyweight of HFHS diet mice was increased compared with normal chow mice during the ninth to 18th week of the experiment, while OCA supplementation showed no influence on body weight. The serum TC levels were increased in mice with an 18-week HFHS diet and reduced by OCA treatment (Figure 1B). The GTT results showed that supplementation with OCA decreased the elevated fasting glucose in HFHS diet mice (Figure 1C). The HFHS diet mice with ABX also showed improvement in serum glucose levels when compared with those with vehicles (Figure 1C,D). Additionally, the serum TG levels were decreased in HFHS diet mice compared with normal chow mice (Figure 1B), accompanied by increased serum levels of ALT and AST (Figure S3A). Since circulating TG levels were defined by the hepatic secretion rate of TG [42] and liver diseases like NASH could decrease the lipid outflow from the liver [43], the decreased serum TG may result from liver injury. The OCA, especially the ABX supplementation, significantly decreased serum levels of ALT and AST (Figure S3A), and ABX supplementation also significantly increased the serum TG levels (Figure 1B). These results supported that gut dysbiosis contributed to MDs in HFHS diet mice, which could be improved by OCA supplementation.

Meanwhile, we assessed the behavior performance of mice in the 18th week. For parameters reflecting locomotor activity, the total distance and mean speed in OF test were unchanged in HFHS diet mice with or without OCA when compared with normal chow mice (Figure 1E,F). Excitedly, the time spent in the central arena of the OF was decreased in HFHS diet mice with the vehicle but unchanged in those with OCA or ABX when compared with normal chow mice (Figure 1G). These findings indicated that gut microbiota was involved in the pathogenesis of anxiety in HFHS diet-induced MDs mice, which could be inhibited by OCA treatment. The training and probe trials of MWM tests showed that HFHS diet mice with or without OCA or ABX intervention displayed no deficit in spatial learning and memory at the 18th week (Figure S4A,B).

**Figure 2:**
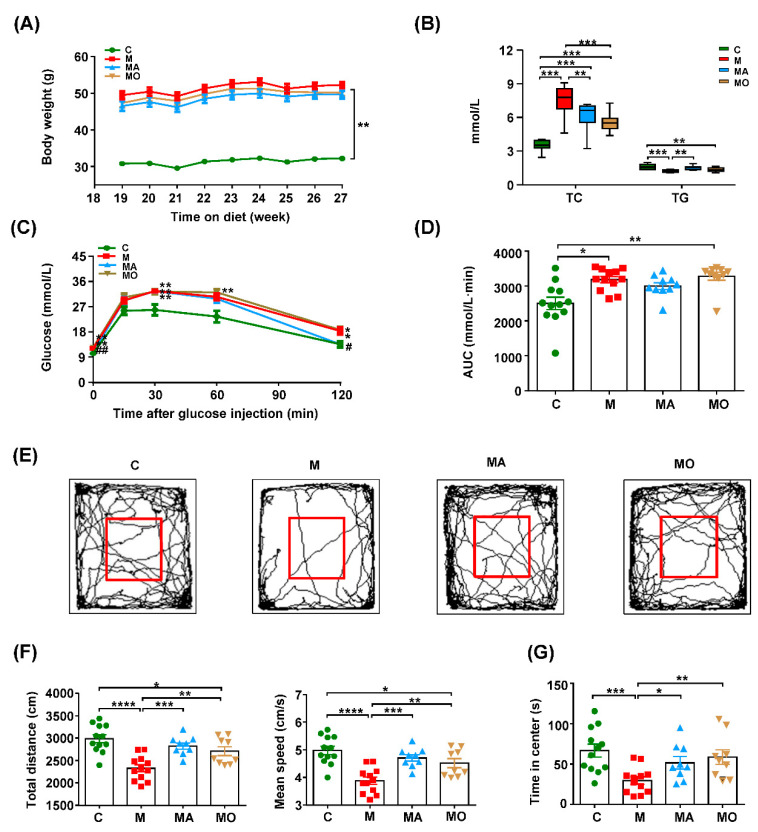
OCA supplementation inhibited the progression of gut microbiota-mediated anxiety in HFHS diet mice. The anxiety levels were increased with the severity of MDs in HFHS diet mice, which were inhibited by OCA or ABX treatment. (**A**–**D**) The body weight, serum TC and TG, GTT and the corresponding AUC were evaluated at the 27th week. (**E**) The representative trace graphs of the OF test at the 27th week. The parameters of the OF test reflecting (**F**) locomotor activity and (**G**) anxiety-like behavior are depicted. Note: data are given as mean ± SEM or medians with range. *n*: (**A**–**G**) 8–12 per group. (**A**,**C**) * *p* < 0.05; ** *p* < 0.01 compared with normal chow mice, # *p* < 0.05; ## *p* < 0.01 compared with HFHS diet mice with vehicle. (**B**,**D**,**G**) * *p* < 0.05; ** *p* < 0.01; *** *p* < 0.001; **** *p* < 0.0001. Groups: C, mice with normal chow; M, mice with HFHS diet; MA, ABX-treated HFHS diet mice; MO, OCA-treated HFHS diet mice. Abbreviations: HFHS, high-fat, high-sugar; MDs, metabolic disorders; ABX, antibiotics; OCA, obeticholic acid; TC, total cholesterol; TG, triglyceride; GTT, glucose tolerance test; AUC, area under the curve; SEM, standard error of mean; OF, open field.

To further confirm the anxiolytic effect of OCA on the progression of MDs-related anxiety, we continued the treatments of OCA to HFHS diet mice until the 27th week. As shown in Figure 2A,B, HFHS diet mice developed severe MDs at the 27th week indicated by obviously increased body weight, elevated serum TC levels and decreased TG levels. Moreover, the GTT revealed impaired glucose metabolism of HFHS diet mice (Figure 2C,D). The OCA or ABX treatment significantly decreased the serum TC levels (Figure 2B). Meanwhile, ABX-treated mice showed an increase of serum TG levels and a decrease of glucose levels at zero and 120 min after glucose injection compared with HFHS diet mice with vehicle (Figure 2B,C). Additionally, OCA or ABX treatment decreased the serum levels of ALT and AST in HFHS diet mice (Figure S3B). As shown in Figure S3C, 27-week HFHS diet-induced NASH indicated by H&E staining of steatosis, hepatocyte ballooning and accumulated inflammatory cells in the liver, which were ameliorated by OCA or ABX treatment.

Accompanied with the severe MDs, HFHS diet mice at the 27th week presented with reduced locomotor activity indicated by the obviously decreased total distance and mean speed in the OF tests compared with normal chow mice, which were reversed by OCA or ABX treatment (Figure 2E,F). Compared with normal chow mice, the markedly decreased central visit time indicated the increased levels of anxiety in HFHS diet mice with severe MDs (Figure 2E,G). The ABX treatment significantly inhibited the anxiety-like behavior of HFHS diet mice (Figure 2E–G). Notably, OCA treatment tended to increase more central visit time of HFHS diet mice than ABX, indicating its better anxiolytic effects (Figure 2E–G). Similar to the results of the 18th week, HFHS diet mice displayed no cognitive deficit at the 27th week indicated by the unchanged MWM learning AUC and time spent in the target quadrant of the probe trail among normal chow mice and HFHS diet mice with or without OCA or ABX (Figure S4C,D). Together, these findings suggested that gut microbiota-mediated the development of anxiety in HFHS diet-induced MDs mice, and OCA supplementation was sufficient to improve MDs and inhibited the related anxiety-like behavior.

### 3.2. OCA Supplementation Ameliorated Gut Microbiota-Mediated Microgliosis in HFHS Diet Mice

**Figure 3:**
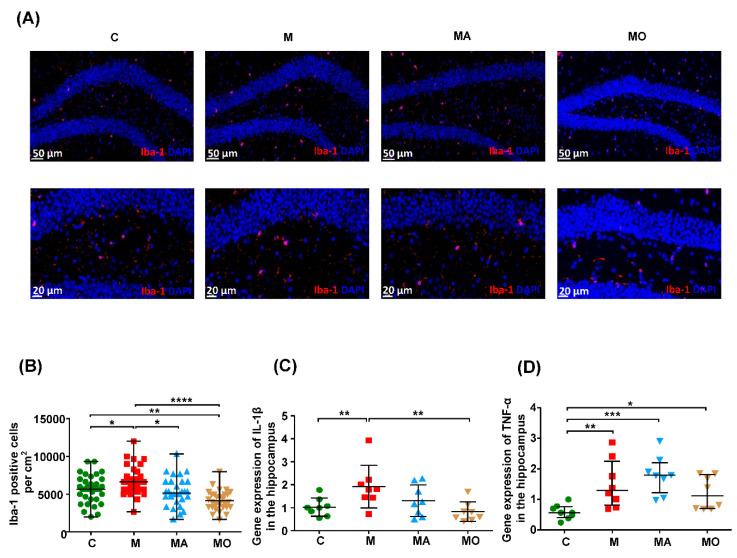
OCA supplementation ameliorated gut microbiota-mediated microgliosis in the hippocampus of HFHS diet mice. HFHS diet mice were characterized with increased microglial cells in the hippocampus and were reduced by ABX treatment. OCA supplementation significantly reversed the microgliosis and proinflammatory cytokines expression in the hippocampus of HFHS diet mice. (**A**) Representative immunofluorescence staining of microglia (Iba-1 positive cells) in the hippocampus. (**B**) Density of Iba-1 positive cells in the hippocampus. (**C**,**D**) The gene expression of proinflammatory IL-1β and TNF-α in the hippocampus. *n*: (**A**–**D**) 5–8 per group. Note: data are given as mean ± SEM. * *p* < 0.05; ** *p* < 0.01; *** *p* < 0.001; **** *p* < 0.0001. Groups: C, mice with normal chow; M, mice with HFHS diet; MA, ABX-treated HFHS diet mice; MO, OCA-treated HFHS diet mice. Abbreviations: HFHS, high-fat high-sugar; qRT–PCR, quantitative real-time polymerase chain reaction; SEM, standard error of the mean; OCA, obeticholic acid; ABX, antibiotics.

Since neuroinflammation was considered as the driving force of anxiety [44], we analyzed the number of microglia and the expression of proinflammatory cytokines in the hippocampus of mice after sacrificing at the end of the 27th week. As shown in Figure 3A–D, the microglial cells were significantly increased, and the expression of IL-1β and TNF-α were also higher in the hippocampus of HFHS diet mice compared with normal chow mice. The OCA treatment significantly decreased the number of microglia and the expression of IL-1β in the hippocampus of HFHS diet mice (Figure 3A–C). The ABX intervention also reduced hippocampal microgliosis (Figure 3A,B). These findings indicated that gut microbiota mediated the increased microglial cells in the hippocampus of HFHS diet mice, and OCA treatment could suppress the microgliosis and microglial activation.

### 3.3. OCA Supplementation Improved Gut Microbiota-Mediated Leaky Gut and Endotoxemia in HFHS Diet Mice

**Figure 4:**
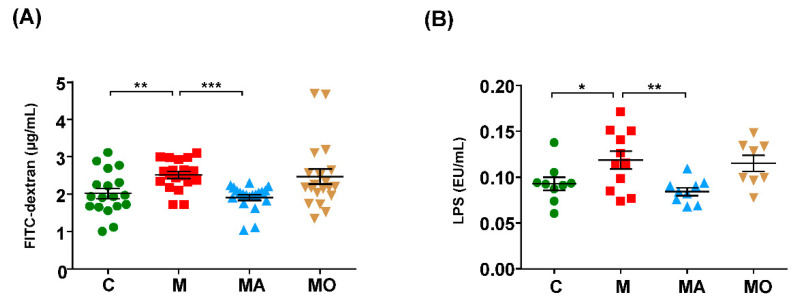
OCA supplementation restored the gut microbiota-mediated increased intestinal permeability and endotoxemia in HFHS diet mice. HFHS diet mice displayed leaky gut and endotoxemia and were reversed by ABX supplementation. OCA treatment restored the intestinal permeability and serum LPS concentration to a normal level. (**A**) The intestinal barrier was evaluated according to the serum concentration of FITC-dextran. (**B**) The serum LPS levels were measured. Note: data are given as mean± SEM. *n*: (**A**–**B**) 8–12 per group. * *p* < 0.05; ** *p* < 0.01; *** *p* < 0.001. Groups: C, mice with normal chow; M, mice with HFHS diet; MA, ABX-treated HFHS diet mice; MO, OCA-treated HFHS diet mice. Abbreviations: HFHS, high-fat high-sugar; ABX, antibiotics; OCA, obeticholic acid; LPS, lipopolysaccharide.

It is well known that microbial component LPS could induce neuroinflammation [13], and intestinal integrity is critical to prevent endotoxemia. Thus, we investigated the effect of OCA on intestinal integrity. As shown in Figure 4A,B, compared with normal chow mice, HFHS diet mice showed increased intestinal permeability and serum LPS levels. OCA treatment restored the intestinal permeability and serum LPS to levels not statistically different from normal chow mice (Figure 4A,B). The ABX supplementation significantly decreased the intestinal permeability and serum LPS levels in HFHS diet mice (Figure 4A,B). These findings indicated that gut dysbiosis contributed to the increased intestinal permeability and endotoxemia in HFHS diet mice, which could be partly restored by OCA treatment.

### 3.4. OCA Supplementation Modified Microbial Composition in HFHS Diet Mice

**Figure 5:**
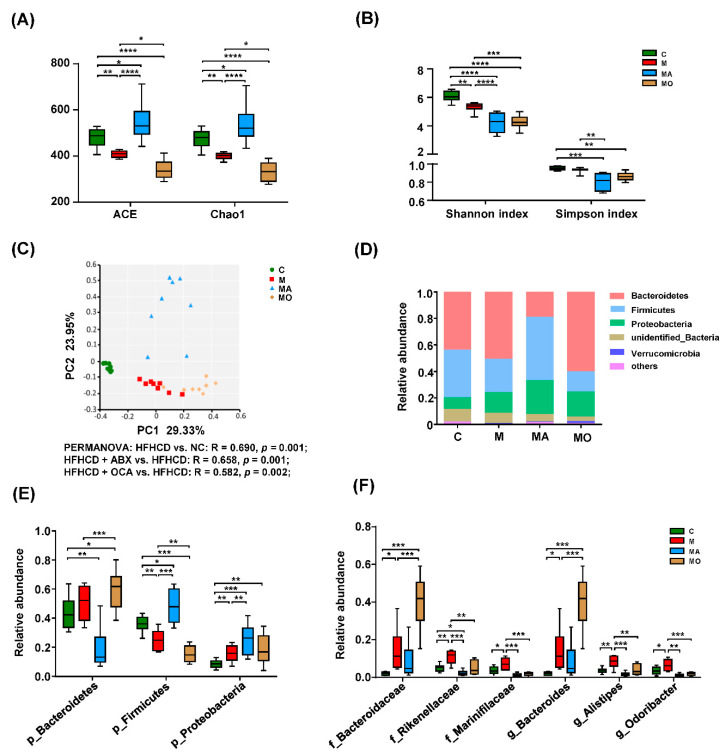
OCA supplementation modified the microbial community in HFHS diet mice. (**A**,**B**) The α-diversity indexes reflecting microbial richness and diversity were calculated. (**C**) The β-diversity indicated the differential microbial community among normal chow mice and HFHS diet mice with or without OCA or ABX treatment. The predominant phyla (**D**) and OCA-affected microbes at (**E**) phylum and (**F**) family and genus levels. Note: data are given as mean± SEM or medians with range. *n*: (**A**–**F**) 8 per group. * *p* < 0.05; ** *p* < 0.01; *** *p* < 0.001; **** *p* < 0.001. Groups: C, mice with normal chow; M, mice with HFHS diet; MA, ABX-treated HFHS diet mice; MO, OCA-treated HFHS diet mice. Abbreviations: HFHS, high-fat high-sugar; PCoA, principal coordinates analysis; ABX, antibiotics; OCA, obeticholic acid; p, phylum; f, family; g, genus.

The α-diversity results of ACE and Chao l showed that the microbial richness was decreased in HFHS diet mice and the Shannon index results indicated that the microbial diversity of HFHS diet mice were reduced compared with normal chow mice (Figure 5A,B). The OCA treatment significantly reduced the microbial richness and diversity of HFHS diet mice, evidenced by the decrease of ACE, Chao 1 and Shannon index compared with those with vehicle (Figure 5A,B). The PCoA plots based on Bray–Curtis metric showed that the IM of HFHS diet mice formed a cluster distinct from those with normal chow (PERMANOVA, R = 0.690, *p* = 0.001), and the OCA supplementation showed a shift in the microbial community compared to HFHS diet mice with vehicle (PERMANOVA, R = 0.582, *p* = 0.002) (Figure 5C). These findings revealed that OCA supplementation modulated the microbial community of HFHS diet mice.

To identify the microbes regulated by OCA supplementation, we further analyzed the predominant microbes with greater than 1% representation in any of the groups. As shown in Figure 5D, the major phyla of normal chow and HFHS diet mice were Bacteroidetes, Firmicutes and Proteobacteria. The HFHS diet mice displayed an increase in Proteobacteria and a decrease in Firmicutes compared with normal chow mice, and the relative abundance of Bacteroidetes showed an increased trend in HFHS diet mice (Figure 5E). In contrast, OCA did not overturn the influence of the HFHS diet on these phyla (Figure 5E). At family and genus levels, totally six predominant microbes were affected by OCA supplementation (Figure 5F). Consistent with the trend at the phylum level, Bacteroidaceae, Rikenellaceae, Marinifilaceae, *Bacteroides*, *Alistipes* and *Odoribacter* from Bacteroidetes were significantly increased in HFHS diet mice compared with normal chow mice (Figure 5F). The Bacteroidaceae and *Bacteroides* were also significantly increased in OCA-treated HFHS diet mice compared with those with vehicle (Figure 5F). Importantly, the neuropsychiatric disorders-related Marinifilaceae and its genera *Odoribacter* were reversed by OCA, and OCA treatment significantly decreased the known anxiety-related microbes, including Rikenellaceae and its genera *Alistipes* in HFHS diet mice (Figure 5F). The ABX supplementation also altered the microbial community of HFHS diet mice indicated by the increased ACE, Chao 1 and the decreased Shannon index, as well as the β-diversity results (PERMANOVA, R = 0.658, *p* = 0.001) (Figure 5A–C). In addition, the relative abundance of Firmicutes and Proteobacteria were increased in ABX-treated mice compared with HFHS diet mice with vehicle (Figure 5E). Similar to OCA supplementation, the ABX treatment also depleted those microbes related to neuropsychiatric disorders, including anxiety in HFHS diet mice (Figure 5F). These findings suggested that OCA may prevent anxiety in HFHS diet mice by modulating microbial communities, especially anxiety-related microbes.

### 3.5. OCA Supplementation Improved Gut Microbiota-Mediated Metabolic Disturbance in HFHS Diet Mice

**Figure 6:**
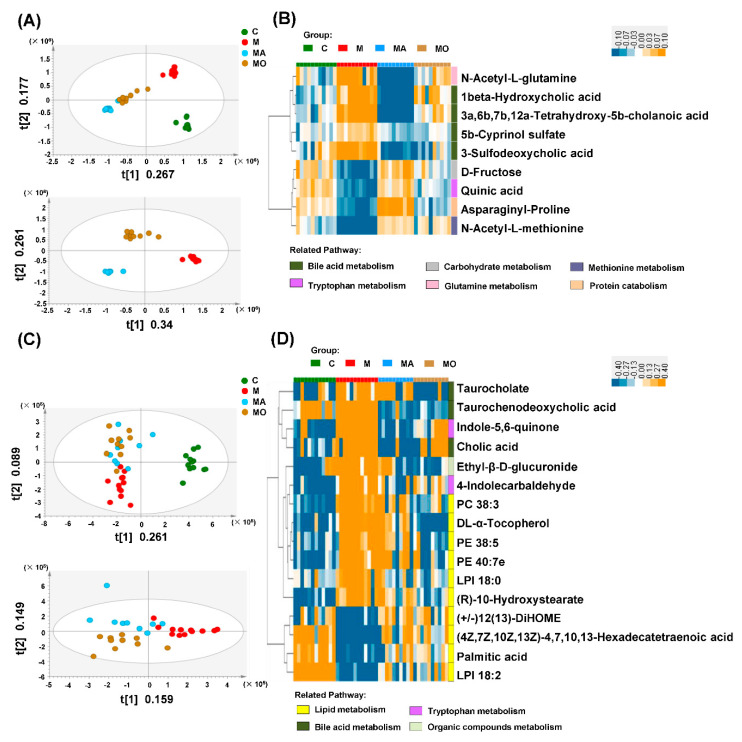
OCA supplementation improved gut microbiota-mediated metabolic disturbance in HFHS diet mice. The OCA supplementation reversed disordered fecal and serum metabolic profiles in HFHS diet mice, and the OCA-modified metabolites were also improved by ABX treatment. PLS-DA models based on data of (**A**) fecal and (**C**) serum metabolomics. The clustering heat map of the differential metabolites improved by OCA and ABX supplementation in (**B**) feces and (**D**) serum of HFHS diet mice. (The abscissa of the clustering heat map corresponds to the group, and the ordinate of different color modules corresponds to related pathways of metabolites. The colors from blue to orange indicate the relative concentration of serum metabolites in each sample). Note: *n*: (**A**–**D**) 10–12 per group. Groups: C, mice with normal chow; M, mice with HFHS diet; MA, ABX-treated HFHS diet mice; MO, OCA-treated HFHS diet mice. Abbreviations: HFHS, high-fat high-sugar; ABX, antibiotics; OCA, obeticholic acid; PLS-DA, partial least-squares-latent structure discriminate analysis; UHPLC, ultra-high-performance liquid chromatography; MS, mass spectrometry.

The effect of OCA on microbial composition raised our interest to investigate its influence on the metabolites derived from gut microbiota. As shown in Figure 6A, the PLS-DA model based on high-throughput fecal metabolomics data showed obvious differences between mice with HFHS diet and normal chow, and OCA supplementation effectively shifted fecal metabolic profiles in HFHS diet mice (Figure 6A). To determine the key microbial metabolites improved by OCA, the VIP values in PLS-DA plotting were computed, and finally, nine biologically significant metabolites were identified (Figure 6B). Notably, four of these nine metabolites reversed by OCA, including 1beta-hydroxycholic acid, 3a,6b,7b,12a-tetrahydroxy-5b-cholanoic acid, 5b-cyprinol sulfate and 3-sulfodeoxycholic acid, were involved in BAs metabolism (Figure 6B). Meanwhile, the decreased quinic acid belonging to tryptophan metabolites in HFHS diet mice was reversed by OCA treatment (Figure 6B). OCA also improved the disturbance in protein and amino acid metabolism (*N*-acetyl-L-glutamine, asparaginyl-proline and *N*-acetyl-L-methionine) and carbohydrate metabolism (D-fructose) of HFHS diet mice (Figure 6B). The improvement of these nine fecal metabolites was also observed in ABX-treated HFHS diet mice (Figure 6B), indicating that OCA supplementation modulated the metabolic function of gut microbiota.

We further analyzed the serum metabolomic profiles to study the influence of OCA on circulating metabolites. Similar to the results in feces, the HFHS diet mice exhibited differential metabolomics profiles from normal chow mice, and OCA supplementation altered the serum metabolites of HFHS diet mice (Figure 6C). A total of 16 metabolites improved by OCA treatment were identified, and these metabolites were annotated to four metabolic pathways of lipids, bile acids, tryptophan and organic compounds (Figure 6D). The serum levels of microbiota-related metabolites, including BAs (TCA, taurochenodeoxycholic acid (TCDCA) and cholic acid (CA)), and tryptophan metabolites (indole-5, 6-quinone and 4-indolecarbaldehyde) were increased in HFHS diet mice compared with normal chow mice and reduced by OCA supplementation (Figure 6D). The HFHS diet mice were also characterized by enriched metabolites in lipid metabolism, including phosphatidylcholine (PC), phosphatidylethanolamine (PE), long-chain fatty acid ((R)-10-hydroxystearate) and DL-α-tocopherol in vitamin E family and organic compounds (ethyl-β-D-glucuronide), which were decreased by OCA treatment (Figure 6D). While lipids metabolites pointed to the metabolism of polyunsaturated fatty acid (PUFA) ((4Z,7Z,10Z,13Z)-4,7,10,13-hexadecatetraenoic acid), linoleic acids ((+/−) 12(13)-DiHOME) and palmitic acid was decreased in HFHS diet mice compared with normal chow mice and increased in OCA-treated mice (Figure 6D). The HFHS diet mice also showed disturbance of lysophospholipid metabolism indicated by the increased LPI 18:0 and decreased LPI 18:2, which were improved by OCA (Figure 6D). Similarly, removing gut microbiota with ABX also improved the serum metabolomics profiles in HFHS diet mice (Figure 6C,D), indicating these circulating metabolites were primarily modulated by gut microbiota. Thus, these metabolomics findings evidenced that gut dysbiosis played important roles in metabolic disturbance of HFHS diet mice and suggested the metabolic mechanism of OCA treatment.

### 3.6. OCA-Reversed Bile Acid Linked Metabolic Disturbance to Anxiety in HFHS Diet Mice

**Figure 7:**
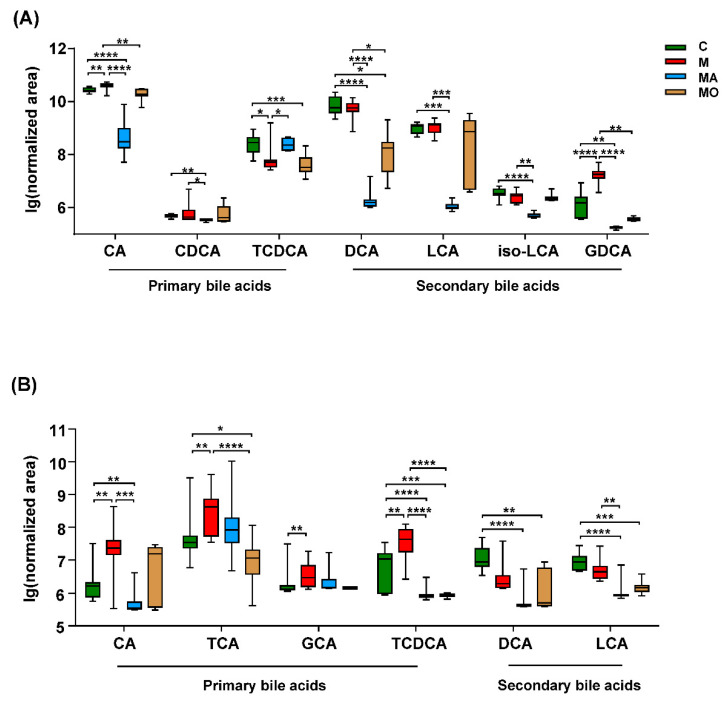
OCA supplementation modulated the gut microbiota-mediated bile acids disorders in HFHS diet mice. OCA supplementation decreased fecal, and serum BAs in HFHS diet mice and these BAs were also reduced by ABX supplementation. (**A**) Seven fecal BAs and (**B**) six serum BAs were detected in each of the mice. Note: data are given as medians with range. *n*: (**A**,**B**) 10–12 per group. * *p* < 0.05; ** *p* < 0.01; *** *p* < 0.001; **** *p* < 0.001. Groups: C, mice with normal chow; M, mice with HFHS diet; MA, ABX-treated HFHS diet mice; MO, OCA-treated HFHS diet mice. Abbreviations: HFHS, high-fat high-sugar; ABX, antibiotics; OCA, obeticholic acid; BAs, bile acids; CA, cholic acid; CDCA, chenodeoxycholic acid; TCDCA, taurochenodeoxycholic acid; DCA, deoxycholic acid; LCA, lithocholic acid; iso-LCA, isolithocholic acid; GDCA, glycodeoxycholic acid; TCA, taurocholate; GCA, glycocholic acid.

Due to the markedly improved BA-related metabolites in both feces and serum of OCA-treated HFHS diet mice, we then focused on BAs metabolites by setting an in-house mass list. As listed in Figure 7A, a total of seven BAs, including three primary BAs and four secondary BAs, were detected in the feces of all mice. Although TCDCA was significantly decreased in HFHS diet mice, the predominant primary bile acid CA and its conjugated secondary bile acid glycodeoxycholic acid (GDCA) were significantly increased in HFHS diet mice compared with normal chow mice and were decreased by OCA supplementation (Figure 7A). Similarly, ABX also decreased CA and GDCA in HFHS diet mice. Additionally, TCDCA was increased, while chenodeoxycholic acid, deoxycholic acid, lithocholic acid and isolithocholic acid were decreased in ABX-treated HFHS diet mice compared with those with vehicle (Figure 7A). These findings indicated that gut dysbiosis led to BAs disorders in HFHS diet mice, which could be partly modified by OCA treatment.

Meanwhile, a total of six BAs, including four primary BAs and two secondary BAs, were detected in the serum of each mice (Figure 7B). The four primary BAs synthesized in the host liver, including CA, TCA, glycocholic acid and TCDCA, were increased in serum of HFHS diet mice, while no significant change was observed in gut-derived-secondary BAs (Figure 7B). Of note, all of these four primary BAs displayed decreased trend in OCA-treated mice, and TCA and TCDCA showed a significant difference when compared with HFHS diet mice with vehicle (Figure 7B). ABX also decreased the serum BAs especially the CA and TCDCA, in HFHS diet mice (Figure 7B). These metabolomics results supported a direct link among OCA treatment, microbial BAs metabolism and host BAs profiles in HFHS diet mice, suggesting the crucial role of the “microbiota–BAs axis” in mediating the beneficial effects of OCA.

**Figure 8:**
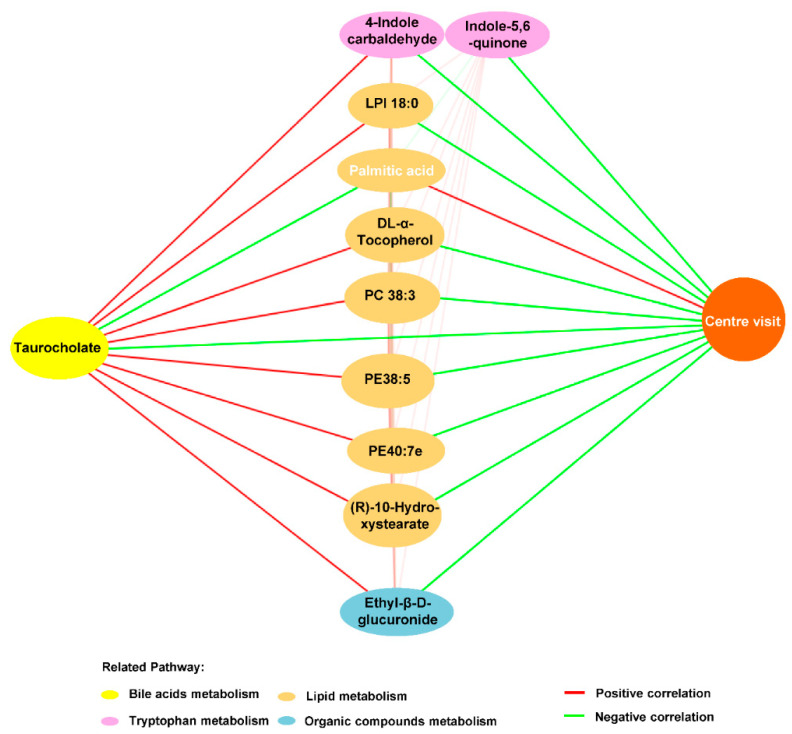
OCA-reversed bile acid taurocholate linked disordered serum metabolites to anxiety-like behavior in HFHS diet mice. Correlation network exhibited that microbial metabolites, including bile acid taurocholate and indole derivatives of tryptophan metabolites, linked the metabolic disturbance to anxiety in HFHS diet mice, which were improved by OCA supplementation. Note: The serum metabolites are color-coded according to the KEGG pathway mapping results, and metabolites marked with black color are increased, and white are decreased in HFHS diet mice. Positive correlations are illustrated as red lines and negative correlations as green lines. Abbreviations: HFHS, high-fat high-sugar; OCA, obeticholic acid; KEGG, Kyoto Encyclopedia of Genes and Genomes.

To further identify the interplay across serum metabolites, including BAs, and the anxiolytic effect of OCA, correlation analysis between the OCA-improved serum metabolites and anxiety-like behavior was performed. Totally, 11 metabolites annotated to four pathways were selected to build the correlation network. As exhibited in Figure 8, the metabolic changes, including 10 increased serum metabolites reflecting enhanced BAs metabolism, tryptophan metabolism, lipid metabolism of lysophospholipid, vitamin E, PE, PC, and long-chain fatty acid (R)-10-hydroxystearate, and organic compounds metabolism in HFHS diet mice were negatively associated with central visit time in the OF test (Figure 8). Conversely, the decreased palmitic acid in HFHS mice was positively associated with time spent in the central arena of the OF (Figure 8). Interestingly, the microbial metabolites, including primary bile acid TCA and two indole derivatives of tryptophan metabolites, were related to the majority of anxiety-related metabolites; especially the TCA was the core metabolite correlated with all of these metabolic perturbations (Figure 8). Together, these findings suggested that the anxiolytic effects of OCA may be mediated by modulating “microbiota–BAs–brain axis”.

## 4. Discussion

Although anxiety has become a health endanger disease worldwide, there remains no clear pathogenesis or mechanistically new drugs for anxiety disorders in the past two decades [1]. Previous clinical trials have suggested OCA can improve MDs [20]; however, whether OCA supplementation can inhibit anxiety in association with MDs remain unknown. Here, we found that the anxiety of HFHS diet-induced MDs mice could be improved by OCA treatment via “microbiota–BAs–brain axis”, profiting from a comprehensive study on behavioral performance, brain tissue histopathological alteration, the shift of microbial community, and network analysis of metabolomics profiling.

In this study, we used the HFHS diet to mimic the palatable ‘‘junk” foods of excess fat and sugar and found the presence of anxiety-like behaviors and MDs in HFHS diet fed-mice at a similar pace. Additionally, the levels of anxiety were exacerbated with the severity of MDs. In agreement with the previous few studies [45,46], our studies indicated the causal relationship between HFHS diet-induced MDs and anxiety. In line with our findings, Youjun Yang et al. showed that early life HFD-induced obesity programs cognitive functions [13]. Juliane Zemdegs et al. showed that HFD withdrawal reversed MDs and improved anxiety in mice [47]. Our study is consistent with the previous few studies, provided new evidence for the link between the MDs induced by overconsumption of HF/HS foods and common psychological disorders of anxiety.

Notably, OCA treatment reversed the BAs enrichment in feces and serum, inhibited the anxiety-like behavior and significantly improved the MDs in HFHS diet-fed mice. Consistently, we revealed that the OCA decreased the density of microglia and ameliorated the expression of neuroinflammatory cytokines in the hippocampus of MDs mice. These results suggested that the modulation of OCA on neuroinflammation of anxiety would be the promising effective mode for anxiolytics. The results of this study evidenced that OCA inhibited the anxiety-like behavior in HFHS diet-MDs mice, which might be mediated by reducing the neuroinflammation in the hippocampus. The enriched microglia in the hippocampus with a high risk of developing anxiety and the inhibition of microglia could reduce the hyperanxiety [48]. Importantly, the high systematic inflammation is associated with resistance to antidepressants, which are the first-line treatment for anxiety disorders currently [48,49]. Our findings indicated that the inhibition effect of OCA treatment on microglia and neuroinflammatory cytokines in the hippocampus might contribute to the protection against the development of anxiety in HFHS-diet mice. In line with our results, OCA supplementation ameliorated neuroinflammation in mice with autoimmune neurological disease multiple sclerosis [50]. Matthew McMillin et al. showed that intervention with BAs sequestrant cholestyramine reduced proinflammatory cytokine in the cortex of chemical-induced hepatic encephalopathy (HE) mice [51]. These findings supported the potential role of BAs enrichment in the pathogenesis of anxiety in our HFHS diet mice. Based on the previous findings [52,53], we hypothesized that BAs might indirectly promote neuroinflammation and directly influence brain function via crossing the blood–brain barrier and acting on the BAs transports and receptors. In accordance with our results, Guoxiang Xie et al. showed that inhibiting the reabsorption of BAs could decrease the anxiety-like behavior of mice [19]. Supported by several previous findings of the potential role of gut-derived BAs in neuropsychiatric disorders [9,19], the findings of this study, to the best of our knowledge, first, indicated the treatment effect of OCA on anxiety in HFHS-induced MDs mice via modulating the “microbiota–BAs–brain” axis.

Further, we found that the OCA-modulated IM led to the altered serum microbial metabolites and might further contribute to the OCA-alleviating effect on anxiety. Importantly, we found that OCA significantly changed the metabolic profiles of HFHS diet- MDs mice by metabolomics analysis, the major of which were microbial metabolites, especially BAs. These findings suggested BAs as crucial mediators in the gut–brain axis concerning anxiety in HFHS diet-MDs mice. Moreover, the beneficial effects of depleting gut microbiota by ABX treatment on anxiety-like behavior, microgliosis and intestinal barrier integrity in this study demonstrated the key role of microbiota in the anxiety of MDs mice and suggested that the OCA-alleviating neuroinflammation may be mediated by modulating microbiota and microbial metabolites.

Herein, the 18-week OCA treatment restored the intestinal barrier integrity and serum LPS of HFHS-fed MDs mice to the level of normal mice. In agreement with our findings, a previous study showed that OCA increased Gram-positive bacteria and decreased Gram-negative bacteria in feces [54], which was favorable for the production of LPS [55]. LPS was considered the most potent natural inflammation factor from bacteria [56] and demonstrated to cause anxiety symptoms in humans [57]. The animal experiments revealed that LPS activated microglia promoted the production of proinflammatory cytokines in the brain and induced anxiety-like behavior [55], while cotreatment with antibiotic doxycycline could attenuate LPS-induced microglial activation and anxiety in mice [58]. In this study, OCA significantly reduced neuropsychiatric disorders-related bacteria in the HFHS-fed MDs mice, such as Marinifilaceae, Rikenellaceae and its genus *Alistipes*, and these microbes have been reported in the previous model and clinical studies [59,60,61]. For example, the indole-positive *Alistipes* could influence the tryptophan metabolism and disrupt the balance of the serotonergic system [61], which was known to modulate neuronal signals related to anxiety [62]. Our findings highly suggested that the inhibiting effect of OCA on the anxiety-related bacteria may be transmitted from the gut to the brain via the “gut microbiota–brain” axis. Thus, our findings of OCA in improving the ‘leaky gut’ and reducing the LPS-producing bacteria in HFHS-diet MDs mice could partially explain how orally OCA supplementation reduced neuroinflammation in the hippocampus via the ”gut–brain” axis.

Furthermore, network analysis identified OCA-affected microbial metabolites, including bile acid TCA and indole derivatives and lipid metabolites were linked to anxiety-like behavior in MDs mice. The OCA-restored microbial community led to alterations in microbial metabolites, especially BAs in the local intestine and circulation, and the circulating TCA was the core metabolite among the interactions of disordered metabolites and anxiety. The increased plasma level of TCA was reported to be associated with the severity of MDs [8], and inhibiting the TCA-associated sphingosine-1-phosphate receptor 2 signalings could reduce microglia proliferation and neuroinflammation in chemical-induced HE mice [51]. It is noteworthy that metabolites derived from gut microbiota were mediators in achieving the crosstalk between IM and the brain [14]. However, research on the “gut–metabolism–brain axis” in MDs-related anxiety is currently lacking. These findings indicated that TCA in MDs might act as a mediator of gut–brain axis in association with anxiety.

The other important microbial metabolites related to the OCA-alleviating effect on anxiety in HFHS diet-MDs mice were indole derivatives of tryptophan metabolites. The indole derivatives were potential ligands of aryl hydrocarbon receptor (AhR) [14], which is a critical node in linking microbial tryptophan metabolites to brain function [63], and was involved in neuroinflammation [64]. In agreement with the findings of enhanced AhR activation in neuropsychiatric disorders of cognitive impairment of AD patients [65] and depression mice [66], the enriched indole derivatives were found in our HFHS diet-MDs mice and significantly related with the levels of circulating bile acid TCA and anxiety-like behavior.

Additionally, these OCA-improved microbial metabolites were correlated with metabolic disorders of circulating lipid metabolites, such as PC and palmitic acid. PC is both the structural constituent of cell membranes and fulfills a variety of regulatory functions linking to the inflammatory process and cell damage [67]. In line with our findings in the MDs-related anxiety mice, experimental research showed the elevated PC in plasma of mice with anxiety disorders [68], and a Dutch family-based lipidomics study presented the association between plasma PC concentrations and anxiety symptoms [69]. Moreover, we found that OCA reversed the decreased palmitic acid of model mice, which was positively associated with the anxiety-like behavior. In agreement with our findings, palmitic acid has been suggested to benefit anxiety disorders [70,71]. Together, our findings suggested the beneficial effects of OCA on MDs-related anxiety via modulating “microbiota–BAs–brain axis”. Overall, these findings provide evidence that targeting BAs for inhibiting anxiety in MDs may be mediated by TCA and the coordinate disturbance of indole derivatives and lipids.

## 5. Conclusions

In conclusion, this novel study demonstrated IM and its metabolized BAs participated in the MDs-related anxiety development. Importantly, we demonstrated that OCA treatment inhibited the development of anxiety in HFHS diet-MDs mice, whose beneficial effects were associated with the improvement on neuroinflammation by modulating “microbiota–BAs–brain axis”. Our findings provided insights into the crucial role of gut microbiota and metabolites BAs in the development of anxiety in MDs and suggested that targeting on BAs metabolism holds the promise for preventing anxiety. However, future studies are needed to identify the underlying molecular mechanisms in the crosstalk between BAs and the brain.

## Data Availability

The microbiome sequence data are deposited in the National Center for Biotechnology Information (NCBI) Bioproject database with project number PRJNA678884.

## Supplementary Materials

The following are available online at https://www.mdpi.com/2072-6643/13/3/940/s1, Figure S1: Study design, Figure S2: HFHS diet mice developed metabolic disorders at the ninth week, Figure S3: OCA supplementation ameliorated gut microbiota-mediated liver injury of HFHS diet mice, Figure S4: HFHS diet mice showed no cognitive function deficit, Table S1: Sequences of PCR primers, Table S2: Mass list library of 30 bile acids.
