# Supplementary material for: Obeticholic Acid Inhibits Anxiety via Alleviating Gut Microbiota-Mediated Microglia Accumulation in the Brain of High-Fat High-Sugar Diet Mice
Source: Nutrients. 2021 Mar 15;13(3):940. doi: 10.3390/nu13030940 (PMC7999854; doi:10.3390/nu13030940)
Supplement: Supplementary file 1 [file nutrients-13-00940-s001.pdf]

1. Supplementary Tables

Supplementary Table S1. Sequences of PCR primers.

Gene	Forward (5' to 3')	Reverse (5' to 3')
β-actin	CACAGCTGAGAGGGAAATCG	AGTTTCATGGATGCCACAGG
IL-1β	CTTCAGGCAGGCAGTATCACTC	GCAGTTGTCTAATGGGAACGTC
TNF-α	CAGGCGGTGCCTATGTCTC	CGATCACCCCGAAGTTCAGTAG

Abbreviations: PCR, polymerase chain reaction.

Supplementary Table S2. Mass list library of 30 bile acids.

Name	Full name	Formula
T-α-MCA	Tauro-alpha-muricholic acid	$C_{26}H_{45}NO_7S$
T-β-MCA	Tauro-beta-muricholic acid	$C_{26}H_{45}NO_7S$
THCA	Taurohyocholic acid	$C_{26}H_{45}NO_7S$
TUDCA	Tauroursodeoxycholic acid	$C_{26}H_{45}NO_6S$
TCA	Taurocholic acid	$C_{26}H_{45}NO_7S$
GHCA	Glycohyocholic Acid	$C_{26}H_{43}NO_6$
GCA	Glycocholic acid	$C_{26}H_{43}NO_6$
ω-MCA	Omega-muricholic acid	$C_{24}H_{40}O_5$
GUDCA	Glycoursodeoxycholic acid	$C_{26}H_{43}NO_5$
GHDCA	Glycohyodeoxycholic acid	$C_{26}H_{43}NO_5$
α-MCA	Alpha-muricholic acid	$C_{24}H_{40}O_5$
β-MCA	Beta-muricholic acid	$C_{24}H_{40}O_5$
TCDCA	Taurochenodeoxycholic acid	$C_{26}H_{45}NO_6S$
TDCA	Taurodeoxycholic acid	$C_{26}H_{45}NO_6S$
HCA	Hyocholic acid	$C_{24}H_{40}O_5$
ACA	Allocholic acid	$C_{24}H_{40}O_5$
CA	Cholic acid	$C_{24}H_{40}O_5$
GCDCA	Glycochenodeoxycholic acid	$C_{26}H_{43}NO_5$
UDCA	Ursodeoxycholic acid	$C_{24}H_{40}O_4$
HDCA	Hyodeoxycholic acid	$C_{24}H_{40}O_4$
GDCA	Glycodeoxycholic acid	$C_{26}H_{43}NO_5$
nutriCA	Nutriacholic acid	$C_{24}H_{38}O_4$
TLCA	Taurolithocholic acid	$C_{26}H_{45}NO_5S$

12-ketoDCA	12-Ketodeoxycholic acid	C24H38O4
CDCA	Chenodeoxycholic acid	C24H40O4
DCA	Deoxycholic acid	C24H40O4
GLCA	Glycolithocholic acid	C26H43NO4
iso-DCA	Isodeoxycholic acid	C24H40O4
iso-LCA	Isolithocholic acid	C24H40O3
LCA	Lithocholic acid	C24H40O3

2. Supplementary Figure

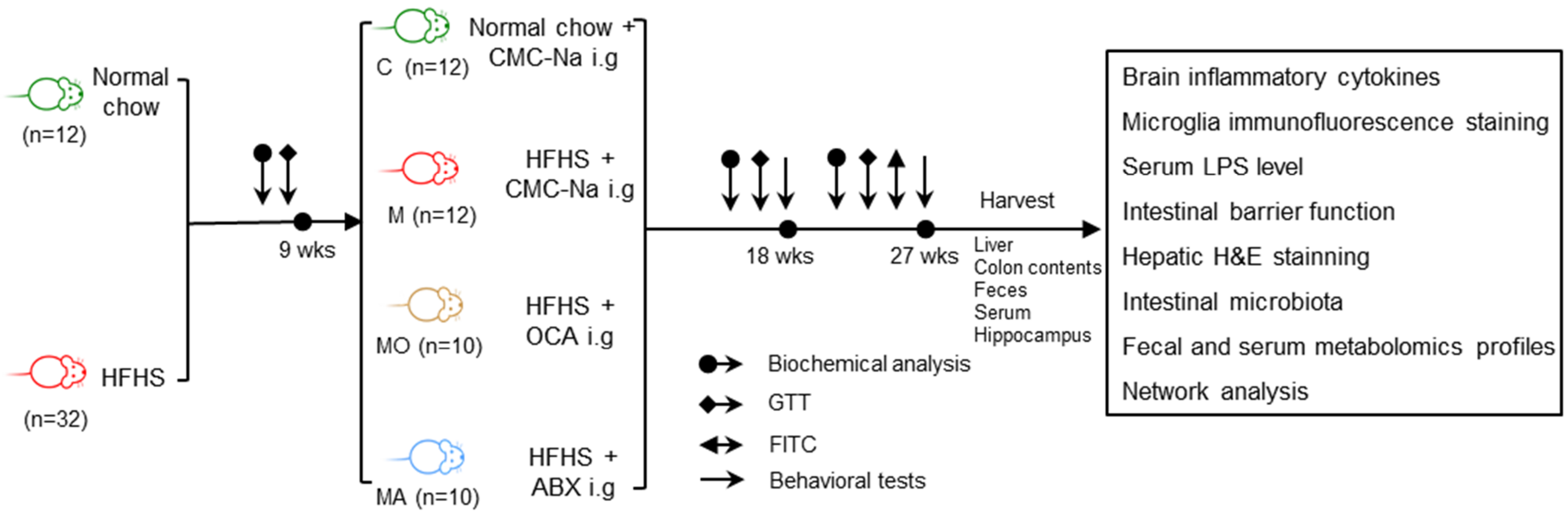

Supplementary Figure S1. Study design. Firstly, the mice were fed with HFHS diet (60% of kcal fat diet and carbohydrates [18.9 g/L sucrose and 23.1 g/L fructose] in drinking water) for nine weeks to induce the MDs indicated by serum lipid parameters and GGT examinations. Then, the HFHS diet mice were divided into three groups including M, MA and MO, and mice with normal chow as control group (C). The mice in MA group were treated with antibiotics cocktail containing1.86 mg ampicillin, 1.86 mg neomycin sulfate, 1.2 mg metronidazole and 0.96 mg vancomycin in 300 μL double distilled water to deplete the commensal bacteria; the mice in MO group were oral gavaged with OCA (2 mg/mL in CMC-Na, 5 mL/kg body weight). During the experimental period, biochemical parameters were analyzed at the 16th and 24th week; the GGT was performed at the 17th and 25th week; the intestinal permeability was assessed using an *in vivo* fluorescein isothiocyanate (FITC)-dextran at the 26th week; the anxiety-like behavior and cognitive function were assessed by performing open field and Morris water maze tests at the 18th and 27th week. All mice were sacrificed at the 27th week, and the effects of ABX and OCA on the expression of proinflammatory cytokines and microgliosis in the hippocampus, intestinal permeability, endotoxemia, liver injury, microbial composition, fecal and serum metabolomics profile were evaluated. The network analysis was also performed to reveal the correlation between disturbed metabolites and anxiety-like behavior. Abbreviations: HFHS, high-fat high-sugar; MDs, metabolic disorders; ABX, antibiotics; OCA, obeticholic acid; GTT, glucose tolerance test; CMC-Na, sodium of carboxymethyl cellulose; LPS, lipopolysaccharide; H&E, hematoxylin and eosin.

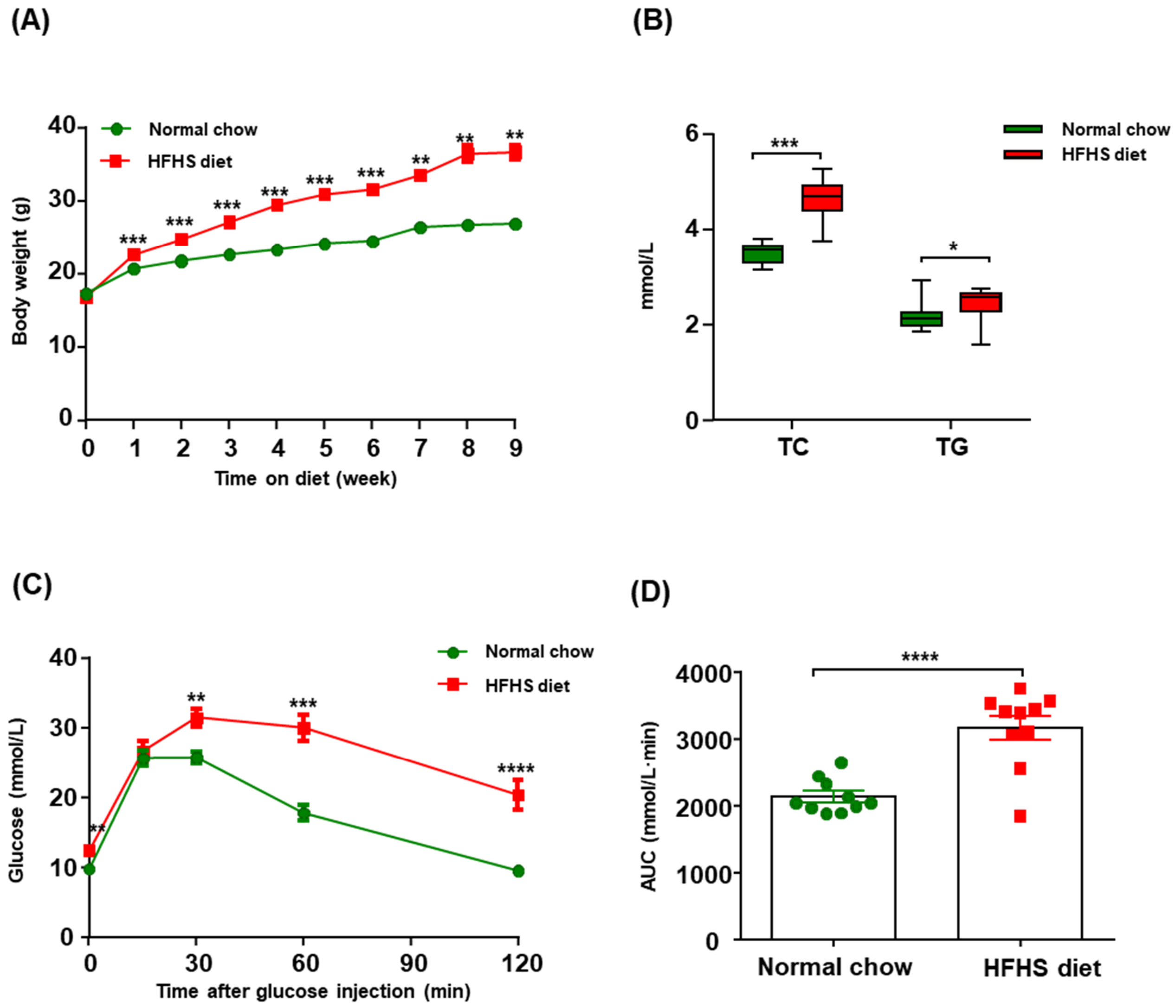
(A)
Normal chow
HFHS diet
Body weight (g)
Time on diet (week)
(B)
Normal chow
HFHS diet
mmol/L
TC
TG
(C)
Normal chow
HFHS diet
Glucose (mmol/L)
Time after glucose injection (min)
(D)
AUC (mmol/L·min)
Normal chow
HFHS diet

Supplementary Figure S2. HFHS diet mice developed metabolic disorders at the ninth week. Mice with nine-week HFHS diet developed MDs indicated by the increased body weight, dyslipidemia and impaired glucose metabolism. (**A**) Body weight of HFHS diet mice were significantly increased compared with mice with normal chow throughout the experimental period. (**B**) The serum lipids parameters (TC, TG) were significantly increased in HFHS diet mice. (**C**,**D**) The GGT and the corresponding AUC revealed impaired glucose metabolism in HFHS diet mice. Note: Data were given as mean± SEM or medians with range. n: (**A**–**D**) 10-12 per group. $^{*}P< 0.05$; $^{**}P< 0.01$; $^{***}P< 0.001$ and $^{****}P< 0.0001$. Abbreviations: MDs, metabolic models; HFHS, high-fat high-sugar; GTT, glucose tolerance test; AUC, area under the curve; TC, total cholesterol; TG, triglyceride; SEM, standard error of mean.

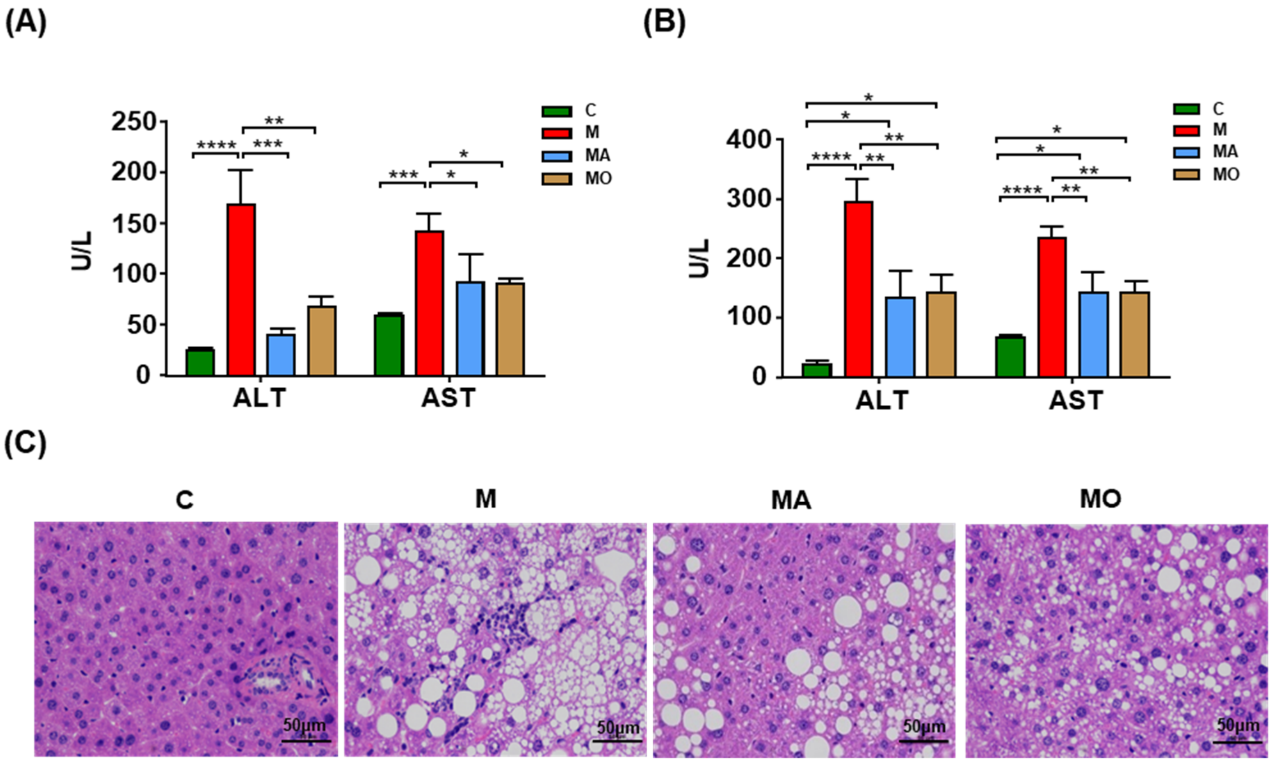
(A)
(B)
(C)
250
200
150
100
50
0
400
300
200
100
U/L
ALT
AST
C
M
MA
MO
****
***
**
*
50μm

Supplementary Figure S3. OCA supplementation ameliorated gut microbiota-mediated liver injury of HFHS diet mice. HFHS diet induced NASH in mice which were ameliorated by OCA and ABX treatment. The serum levels of ALT and AST were increased in mice with (**A**) 19-week and (**B**) 27-week HFHS diet, and were significantly decreased in OCA- and ABX- treated mice. (**C**) H&E staining of the liver showed that mice with 27-week HFHS diet developed NASH indicated by histological alterations including steatosis, hepatocyte ballooning and lobular inflammation, which were alleviated by OCA and ABX supplementation. Note: Data were given as mean± SEM. n: (**A**,**B**) 5 per group. $^{*}P< 0.05$; $^{**}P< 0.01$; $^{***}P< 0.001$ and $^{****}P< 0.0001$. Groups: C, mice with normal chow; M, mice with HFHS diet; MA, ABX-treated HFHS diet mice; MO, OCA-treated HFHS diet mice. Abbreviations: HFHS, high-fat high-sugar; ABX, antibiotics; OCA, obeticholic acid; ALT, alanine aminotransferase; AST, aspartate transaminase; H&E, hematoxylin and eosin; NASH, nonalcoholic steatohepatitis; SEM, standard error of mean.

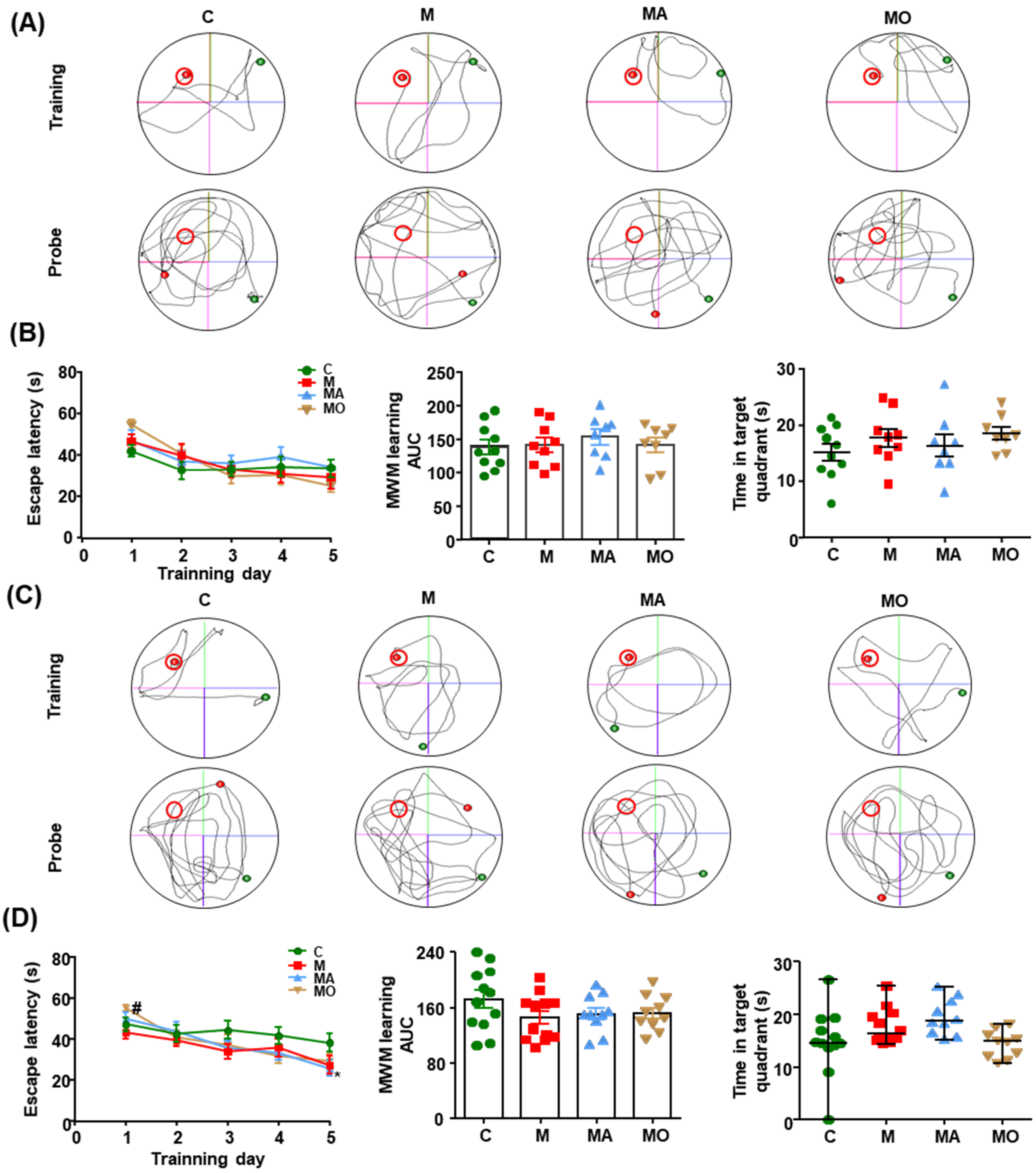

Supplementary Figure S4. HFHS diet mice showed no cognitive function deficit. The cognitive function including spatial learning and spatial memory were evaluated by MWM test. (**A**) The representative trace graphs of swim paths in training and probe trails of the MWM and (**B**) the escape latency and AUC of the training trails, spent in targeted quadrant of the probe trail were recorded in mice with 18-week HFHS diet.

Likewise, (**C**) the representative trace graphs of swim paths in training and probe trails of the MWM and (**D**) the escape latency and AUC of the training trails, spent in targeted quadrant of the probe trail were also evaluated at the 27th week. Note: Data were given as mean± SEM or medians with range. n: (**A**–**D**) 8-10 per group. $^{*}P < 0.05$ compared with normal chow mice; $^{\#}P < 0.05$ compared with HFHS mice with vehicle. Groups: C, mice with normal chow; M, mice with HFHS diet; MA, ABX-treated HFHS diet mice; MO, OCA-treated HFHS diet mice. Abbreviations: HFHS, high-fat high-sugar; ABX, antibiotics; OCA, obeticholic acid; AUC, area under the curve; SEM, standard error of mean; MWM, Morris water maze.
